# Supplementary material for: Unsupervised meta-clustering identifies risk clusters in acute myeloid leukemia based on clinical and genetic profiles
Source: Commun Med (Lond). 2023 May 17;3:68. doi: 10.1038/s43856-023-00298-6 (PMC10192332; doi:10.1038/s43856-023-00298-6)
Supplement: Supplementary file 1 — Supplementary Information [file 43856_2023_298_MOESM1_ESM.pdf]

TruSight Myeloid Sequencing Panel				
ABL1	CEBPA	HRAS	MYD88	SF3B1
ASXL1	CSF3R	IDH1	NOTCH1	SMC1A
ATRX	CUX1	IDH2	NPM1	SMC3
BCOR	DNMT3A	IKZF1	NRAS	SRSF2
BCORL1	ETV6/TEL	JAK2	PDGFRA	STAG2
BRAF	EZH2	JAK3	PHF6	TET2
CALR	FBXW7	KDM6A	PTEN	TP53
CBL	FLT3	KIT	PTPN11	U2AF1
CBLB	GATA1	KRAS	RAD21	WT1
CBLC	GATA2	MLL	RUNX1	ZRSR2
CDKN2A	GNAS	MPL	SETBP1	

Table S1. Summary of the 54 genes targeted by the TruSight Myeloid Sequencing Panel

Transformation algorithm	abbreviation
Principal Component Analysis	PCA
Incremental Principal Component Analysis	IPCA
Sparse Principal Component Analysis	SPCA
Singular Value Decomposition	SVD
Gaussian Random Projection	GRP
Sparse Random Projection	SRP
Multi-Dimensional Scaling	MDS
ISOMAP	ISOMAP
Linear Local Embedding	LLE
Mini-Batch Dictionary Learning	MBDL
Auto-Encoder	AE

Table S2 list of transformation algorithms. Parameters were transferred from an original data space to a model data space by transforming, scaling and dimensionality reduction using eleven transformation algorithms. This way, data was made accessible to unsupervised clustering algorithms while at the same time keeping original Euclidean distances between parameters and reducing dimensionality for model stability.

**Unsupervised clustering algorithm**	**abbreviation**
k-Means	
Two Means	
Agglomerative Clustering	AC
Spectral Clustering	SC
Linkage	
BIRCH	
Gaussian Mixture	GM
OPTICS	
MeanShift	
DBSCAN	
Affinity Propagation	AP

Table S3 list of clustering algorithms. Based on intermediate data models that were generated using transformation algorithms, eleven unsupervised clustering algorithms were implemented to cluster patients according to their similarities and differences regarding clinical, laboratory and genetic parameters.

package	version
click	7.1.2
coverage	5.3
flake8	3.8.4
matplotlib	3.3.2
missingno	0.4.2
numpy	1.18.5
numpydoc	1.1.0
pandas	1.1.4
pytablewriter	0.58.0
python-dotenv	0.15.0
scikit-learn	0.23.2
seaborn	0.11.0
sklearn-pandas	2.0.2
Sphinx	3.3.0
sphinx-rtd-theme	0.5.0
PyYAML	5.3.1
xgboost	1.2.1
yellowbrick	1.2
imbalanced-learn	0.7.0
sphinxcontrib-images	0.9.2
scikit-optimize	0.8.1
tune-sklearn	0.1.0
ray[tune]	1.0.1.

Table S4 Python packages used for model building

Model	F1	Precision	Recall	AUROC
naive Bayes	0,73	0,78	0,74	0.41
Gradient Boosting	0,88	0,88	0,88	0.98
Random Forest	0,89	0,89	0,89	0.97
Logistic Regression	0,92	0,92	0,92	0.99

Table S5 Comparison of supervised model performance for cluster label assignment on the test set of the original cohort using a 80:20 train-test-split

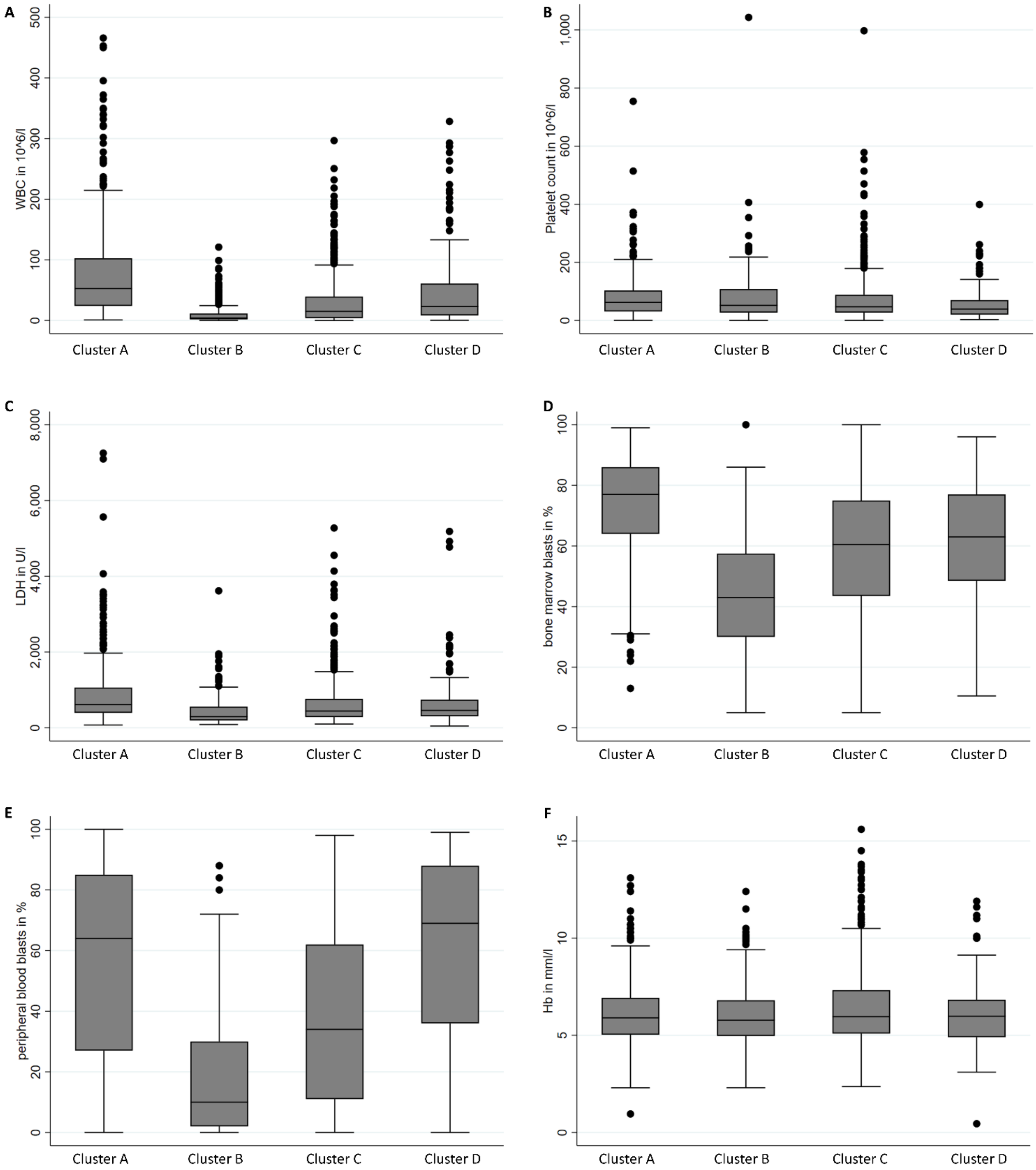

Figure S1. Distribution of continuous variables over clusters. White blood cell count (WBC), platelet count, lactate dehydrogenase (LDH), bone marrow blast count, peripheral blast count and hemoglobin (Hb) are graphed to display varying levels between clusters. Boxplot: bold horizontal line = median; box = interquartile range (IQR, i.e. 25th to 75th percentile); lower

whisker = Q1 – 1.5 * IQR; upper whisker = Q3 + 1.5 * IQR; dots = outliers. $n_{Cluster\ A}$ = 424 patients, $n_{Cluster\ B}$ = 256 patients, $n_{Cluster\ C}$ = 536 patients, $n_{Cluster\ D}$ = 167 patients.

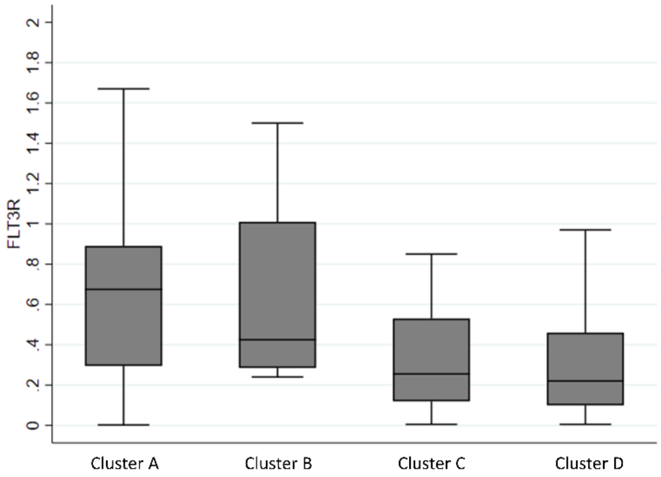

Figure S2. Differences in *FLT3*-ITD ratio between clusters. Boxplot: bold horizontal line = median; box = interquartile range (IQR, i.e. 25th to 75th percentile); lower whisker = Q1 – 1.5 * IQR; upper whisker = Q3 + 1.5 * IQR; dots = outliers. $n_{Cluster\ A}$ = 260 patients with *FLT3*-ITD, $n_{Cluster\ B}$ = 5 patients with *FLT3*-ITD, $n_{Cluster\ C}$ = 32 patients with *FLT3*-ITD, $n_{Cluster\ D}$ = 25 patients with *FLT3*-ITD

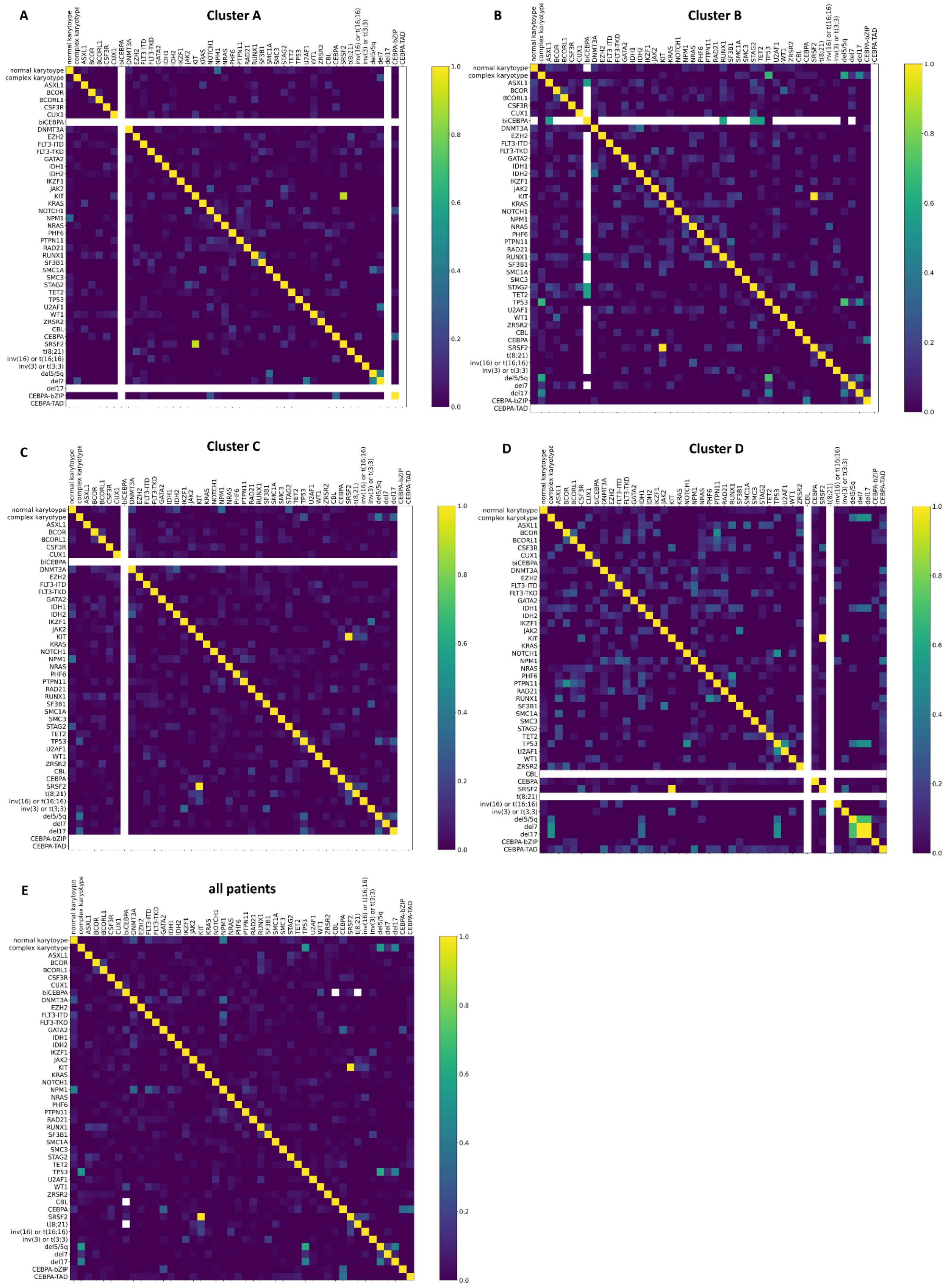

Figure S3. Heatmaps of molecular and cytogenetic alterations within clusters and for the

entire cohort of AML patients. Differences in both cytogenetic and molecular genetic alterations are displayed within each cluster (A-D) and within the entire patient cohort for reference (E).
