# Supplementary material for: Unsupervised meta-clustering identifies risk clusters in acute myeloid leukemia based on clinical and genetic profiles
Source: Commun Med (Lond). 2023 May 17;3:68. doi: 10.1038/s43856-023-00298-6 (PMC10192332; doi:10.1038/s43856-023-00298-6)
Supplement: Supplementary file 14 — Description of Additional Supplementary Files [file 43856_2023_298_MOESM14_ESM.pdf]

Description of Additional Supplementary File

File Name: Supplementary Data 1

Description: Summary of clinical trial protocols and treatment regimens for previous clinical trials that were used to generate patient numbers for clustering and external validation.

File Name: Supplementary Data 2

Description: Available data including clinical data, laboratory values, molecular genetics and cytogenetics used for unsupervised clustering. For the purpose of dimension reduction, features that were very rare (present in less than 1% of patients) were dropped.

File Name: Supplementary Data 3

Description: Missing data for variables included in cluster generation.

File Name: Supplementary Data 4

Description: Variables used for cluster formation. In order to avoid the 'curse of dimensionality', i. e. the destabilizing nature of high-dimensional data sets with only limited data points in them, a feature had to be present in at least 1% of patients to be taken into account by the unsupervised model. Pre-processing of data using this cut-off allowed for stable computations without noise from underrepresented features that would otherwise distort the distribution of data across n dimensions for n features. Further, apart from the 'curse of dimensionality' high values for n make the computation rather inefficient while likely not adding value if only sparse features (<1% present in the patient cohort) are included. The list depicted above constitutes the variables used for cluster generation. Each variable is weighted equally, i. e. no manual interference was undertaken to put additional weight to any of these variables which may potentially have introduced bias and would have been diametrically different from a data-driven rather than hypothesis-driven approach. Lastly, outcome variables were explicitly excluded from cluster generation and only information upon initial diagnosis was used stemming from clinical, laboratory as well as molecular and cytogenetic investigations.

File Name: Supplementary Data 5

Description: Performance metrics for individual clustering and transformation algorithm combinations. Silhouette analysis measures how close a point in a cluster is to points in neighboring clusters. On a scale from -1 to +1, samples that are far away from neighboring clusters will receive a value close to +1 while a value close to 0 means that a sample is close to the decision boundary between two clusters whereas a value close to -1 indicates an error in cluster assignment. The Calinski-Harabasz-Index (also known as the Variance Ratio Criterion) is the ratio between the sum of inter-cluster dispersion and the sum of intra-cluster dispersion. It ranges from 0 to (theoretically) infinity with higher values indicating

higher clustering quality. Lastly, the Davies-Bouldin-Score is an average similarity index that compares each cluster to its most similar cluster. A ratio is formed of intra-cluster distances to inter-cluster distances. The score ranges from 0 to (theoretically) infinity where values close to 0 indicate a higher distance between clusters and thus, better overall cluster quality. For algorithm abbreviations, please see Tables S5-6. #Dim – number of target dimensions; #Cl – number of clusters.

File Name: Supplementary Data 6

Description: Baseline patient characteristics according to risk clusters identified by unsupervised learning. Demographic, clinical and laboratory data are compared between the four risk clusters. Percentages for demographic and clinical parameters refer to the respective cluster. Abbreviations: BM: bone marrow; Hb: hemoglobin; HSCT: hematopoietic stem cell transplantation; IQR: interquartile range; n: number; PB: peripheral blood. Plt: platelet count; WBC: white blood cell count.

File Name: Supplementary Data 7

Description: Molecular genetics and cytogenetics in the four clusters identified by unsupervised learning. Absolute numbers and relative proportions with respect to the individual cluster are displayed for molecular genetic and cytogenetic alterations for each cluster and for the entire patient cohort as a reference.

File Name: Supplementary Data 8

Description: Differences in outcome according to clusters identified by unsupervised learning. Odds ratios (OR) for achievement of complete remission (CR) were obtained using univariable logistic regression. Hazard ratios (HR) for event-free survival (EFS), relapse-free survival (RFS) and overall survival (OS) were obtained using univariable Cox proportional hazard models. 95%-confidence intervals (95%-CI) and adjusted p-values obtained with the Benjamini-Hochberg method are reported for all calculations as well as the interquartile range (IQR) for survival times. Other abbreviations: n: number; n.r.: not reached. (nCluster A = 424 patients, nCluster B = 256 patients, nCluster C = 536 patients, nCluster D = 167 patients)

File Name: Supplementary Data 9

Description: Molecular genetics and cytogenetics in ELN2017 groups within Cluster A. The percentages refer to the number of patients within the ELN2017 subgroup.

File Name: Supplementary Data 10

Description: Molecular genetics and cytogenetics in ELN2017 groups within Cluster B. The percentages refer to the number of patients within the ELN2017 subgroup.

File Name: Supplementary Data 11

Description: Molecular genetics and cytogenetics in ELN2017 groups within Cluster C. The percentages refer to the number of patients within the ELN2017 subgroup.

File Name: Supplementary Data 12

Description: Molecular genetics and cytogenetics in ELN2017 groups within Cluster D. The percentages refer to the number of patients within the ELN2017 subgroup.
