# Supplementary material for: Unsupervised meta-clustering identifies risk clusters in acute myeloid leukemia based on clinical and genetic profiles
Source: Commun Med (Lond). 2023 May 17;3:68. doi: 10.1038/s43856-023-00298-6 (PMC10192332; doi:10.1038/s43856-023-00298-6)
Supplement: Supplementary file 15 — Reporting Summary [file 43856_2023_298_MOESM15_ESM.pdf]

nature portfolio

Corresponding author(s): Jan-Niklas Eckardt

Last updated by author(s): Apr 19, 2023

# Reporting Summary

Nature Portfolio wishes to improve the reproducibility of the work that we publish. This form provides structure for consistency and transparency in reporting. For further information on Nature Portfolio policies, see our Editorial Policies and the Editorial Policy Checklist.

## Statistics

For all statistical analyses, confirm that the following items are present in the figure legend, table legend, main text, or Methods section.

| n/a | Confirmed | |
|---|---|---|
| ☐ | ☒ | The exact sample size (*n*) for each experimental group/condition, given as a discrete number and unit of measurement |
| ☐ | ☒ | A statement on whether measurements were taken from distinct samples or whether the same sample was measured repeatedly |
| ☐ | ☒ | The statistical test(s) used AND whether they are one- or two-sided<br>*Only common tests should be described solely by name; describe more complex techniques in the Methods section.* |
| ☐ | ☒ | A description of all covariates tested |
| ☐ | ☒ | A description of any assumptions or corrections, such as tests of normality and adjustment for multiple comparisons |
| ☐ | ☒ | A full description of the statistical parameters including central tendency (e.g. means) or other basic estimates (e.g. regression coefficient) AND variation (e.g. standard deviation) or associated estimates of uncertainty (e.g. confidence intervals) |
| ☐ | ☒ | For null hypothesis testing, the test statistic (e.g. *F*, *t*, *r*) with confidence intervals, effect sizes, degrees of freedom and *P* value noted<br>*Give P values as exact values whenever suitable.* |
| ☒ | ☐ | For Bayesian analysis, information on the choice of priors and Markov chain Monte Carlo settings |
| ☒ | ☐ | For hierarchical and complex designs, identification of the appropriate level for tests and full reporting of outcomes |
| ☒ | ☐ | Estimates of effect sizes (e.g. Cohen's *d*, Pearson's *r*), indicating how they were calculated |

*Our web collection on statistics for biologists contains articles on many of the points above.*

## Software and code

Policy information about availability of computer code

| | |
|---|---|
| Data collection | - |
| Data analysis | https://zenodo.org/badge/latestdoi/629506656 |

For manuscripts utilizing custom algorithms or software that are central to the research but not yet described in published literature, software must be made available to editors and reviewers. We strongly encourage code deposition in a community repository (e.g. GitHub). See the Nature Portfolio guidelines for submitting code & software for further information.

## Data

Policy information about availability of data

All manuscripts must include a data availability statement. This statement should provide the following information, where applicable:

- Accession codes, unique identifiers, or web links for publicly available datasets
- A description of any restrictions on data availability
- For clinical datasets or third party data, please ensure that the statement adheres to our policy

Data that was used for initial clustering in this study stems from previously reported multi-center trials (AML96, AML2003, AML60+, and SORAML) of the SAL. Data that was used for external validation stems from previously reported trials of the AMLCG (AMLCG-1999 and AMLCG2008). Further information on patients enrolled in these trials can be obtained from the individual references. Data is available from the corresponding author upon reasonable request. Full public access is

currently not possible due to ongoing retrospective studies of the respective study alliances with currently unpublished results. The underlying data for the figures generated for the purpose of this study can be found in Supplementary Data 7 (Figure 3), Supplementary Data 8 (Figure 4), Supplementary Data 9-12 (Figure 5).

## Human research participants

Policy information about studies involving human research participants and Sex and Gender in Research.

| | |
|---|---|
| Reporting on sex and gender | appropriate terminology was used in the context of clinical trial data |
| Population characteristics | Detailed information on population characteristics are given in Supplementary Data 6 |
| Recruitment | not a prospective clinical trial |
| Ethics oversight | Institutional Review Board of the Technical University Dresden (EK 98032010) and Institutional Review Board of the Ludwig-Maximillians-University Munich (EK427-13) |

Note that full information on the approval of the study protocol must also be provided in the manuscript.

# Field-specific reporting

Please select the one below that is the best fit for your research. If you are not sure, read the appropriate sections before making your selection.

☒ Life sciences ☐ Behavioural & social sciences ☐ Ecological, evolutionary & environmental sciences

For a reference copy of the document with all sections, see nature.com/documents/nr-reporting-summary-flat.pdf

# Life sciences study design

All studies must disclose on these points even when the disclosure is negative.

| | |
|---|---|
| Sample size | retrospective computational analysis on data from 6 combined clinical trials, please see Methods - patient data. |
| Data exclusions | features present in <1% of the sample were excluded from the model to avoid the 'curse of dimensionality'. For an in-depth explanation, please see Methods - Data pre-processing and dimensionality reduction |
| Replication | full code for analysis is available under https://zenodo.org/badge/latestdoi/629506656 |
| Randomization | not a prospective clinical trial |
| Blinding | not a prospective clinical trial |

# Reporting for specific materials, systems and methods

We require information from authors about some types of materials, experimental systems and methods used in many studies. Here, indicate whether each material, system or method listed is relevant to your study. If you are not sure if a list item applies to your research, read the appropriate section before selecting a response.

### Materials & experimental systems

| n/a | Involved in the study | |
|---|---|---|
| ☒ | ☐ | Antibodies |
| ☒ | ☐ | Eukaryotic cell lines |
| ☒ | ☐ | Palaeontology and archaeology |
| ☒ | ☐ | Animals and other organisms |
| ☐ | ☒ | Clinical data |
| ☒ | ☐ | Dual use research of concern |

### Methods

| n/a | Involved in the study | |
|---|---|---|
| ☒ | ☐ | ChIP-seq |
| ☒ | ☐ | Flow cytometry |
| ☒ | ☐ | MRI-based neuroimaging |

## Clinical data

Policy information about clinical studies

All manuscripts should comply with the ICMJE guidelines for publication of clinical research and a completed CONSORT checklist must be included with all submissions.

| | |
|---|---|
| Clinical trial registration | Data was taken from previously published clinical trials. Please see the respective clinicaltrial.gov identifiers in Supplementary Data 1 |
| Study protocol | not applicable |
| Data collection | not applicable |
| Outcomes | not applicable |
